# Development of a web-based application to improve data collection of antimicrobial utilization in the public health care system in South Africa

**DOI:** 10.1080/21548331.2021.1889213

**Published:** 2021-03-12

**Authors:** D Kruger, NN Dlamini, JC Meyer, B Godman, A Kurdi, M Lennon, M. Bennie, N Schellack

**Affiliations:** a School of Pharmacy, Sefako Makgatho Health Sciences University, Pretoria, South Africa; b Pharmacy, Private Hospital, Pretoria, South Africa; c Department of Pharmacoepidemiology, Strathclyde Institute of Pharmacy and Biomedical Sciences, University of Strathclyde, Glasgow, UK; d School of Pharmaceutical Sciences, Universiti Sains Malaysia, Penang, Malaysia; e Department of Pharmacology, College of Pharmacy, Hawler Medical University, Erbil, Iraq; f Computer and Information Sciences, University of Strathclyde, Glasgow, UK

**Keywords:** Point prevalence survey, antimicrobials, app, mHealth, South Africa

## Abstract

**Objective:**

Determining antimicrobial utilization patterns in hospitals can be a challenge given personnel and resource constraints with paper-based systems. A web-based application (APP) was developed in South Africa to address this, building on a recent point prevalence survey (PPS) using a paper-based system. Consequently, there was a need to test and evaluate the ease of use of a newly developed app and potential time saving versus paper-based methods for PPS. The findings can be used to further refine the APP.

**Methods:**

The developed app was tested in a large academic public hospital in a PPS in South Africa. During data collection, the app was evaluated for functionality on 35 variables and subsequently refined. After data collection, the app was evaluated in terms of its time-saving potential and ease of use.

**Results:**

181 patient’s files were surveyed across 13 wards in the hospital, with the antimicrobial usage findings similar to the previous paper-based study in the same hospital. The median age for males was 45.5 years and 42 years for females. Overall 80 out of 181 (44%) patients received antibiotics. Whilst 38% (12 out of 31) of patients in the adult surgical ward received antimicrobials, the prevalence was the highest (78%) in the pediatric medical wards. All the data collectors were confident in using the app after training and found the tool is not complex at all to use. In addition, the time taken to plan for the study and to collect data was considerably reduced. Reduced time spent in data collection and analysis is important for timely instigation of quality improvement programs in resource limited settings.

**Conclusions:**

All data collectors would recommend the app for future PPSs. Several concerns with data entry were identified, which have now been addressed. The app development has been successful and is now being deployed across South Africa as part of a national PPS as well as wider.

## 1. Introduction

Antimicrobials play a vital role in improving care and reducing morbidity and mortality in patients with infections [1,2]. However, there are growing concerns that the increasing and often inappropriate use of antimicrobials appreciably increases resistance rates, as well as morbidity, mortality, and costs [3–5]. This has resulted in the development of global and national action plans to reduce antimicrobial resistance (AMR) including in South Africa, which are ongoing [6–12].

One strategy to improve the use of antimicrobials in hospitals is the instigation of point prevalence surveys (PPSs) to provide accurate data on current antibiotic utilization and resistance patterns using a standardized methodology to plan future interventions [13–18]. This is particularly important in South Africa as there are concerns with rationale antibiotic prescribing with high rates of AMR [6,19], and a PPS conducted in 2015 in a large tertiary hospital in South Africa showed that 31% of patients were receiving antibiotics, with the majority (83%) of antibiotic prescriptions being empirical [20]. High empiric antibiotic use is not helped by concerns with reliable surveillance tools or methods linking information between pharmacies, prescribers and laboratories in South Africa [6]. In addition, data on antibiotic utilization collected in the Gauteng Province during World Antibiotic Awareness Week in 2015 were not sufficiently robust to make reliable and valid interpretations and recommendations, highlighting the need for valid and reliable surveillance tools to provide a baseline for pertinent quality improvement programs to enhance future antimicrobial use within the public hospitals in South Africa [6,7,21]. As part of the AMR National Strategy Framework in South Africa, one of the domains is the strengthening of antimicrobial consumption surveillance, with a situational analysis on AMR performed by the Global Antibiotic Resistance Partnership (GARP) South Africa in 2011 identifying the urgent need for South Africa to take action against AMR [6].

There have though been existing concerns with the sustainability of the current paper-based PPSs especially in lower and middle income countries (LMICs) given the length of time that can be needed to undertake comprehensive data collection using agreed data collection instruments (DCI); although this is not always the case with less comprehensive DCIs [18,22]. Considerable time taken for data collection can have considerable economic implications in terms of both manpower and costs since the data from a single ward have to be collected in one day, which could be very challenging in large hospitals in countries such as South Africa [11]. This is particularly the case if PPS studies are to be rapidly repeated to address concerns with identified prescribing, including issues of high empiric use, prolonged use of antibiotics to prevent surgical site infections (SSIs) as well as concerns with the delay in switching patients from IV to oral antibiotics where applicable [11,21,23,24]. In addition, the scarcity of pharmacists and other potential data collectors may make it difficult to release these people for any length of time to undertake PPS studies in their hospital. We are also aware of possible data errors through data collection and entry [25].

The use of a web–based application could potentially address these concerns [26]. For the Global PPS of Antimicrobial Consumption and Resistance (Global PPS), a web-based application was used for data-entry, validation and reporting, which allowed the global PPS to stretch over 53 countries and include 335 hospitals. Based on the success and effectiveness of the Global PPS forms with anonymized data collected and entered onto specifically designed databases [13], combined with the challenges experienced with a paper-based data collection tool for PPS studies in LMICs, an app was developed in April 2017 in South Africa based on the paper-based forms developed and refined from the PPS study in Botswana [11,18,21,26]. The presence of Human Immunodeficiency Virus (HIV), tuberculosis (TB), malaria, and malnutrition was included as variables in our PPS studies, similar to the studies in Botswana and Zimbabwe, as they will influence potential antimicrobial use in sub-Saharan Africa. This is different from the European Center for Disease Prevention and Control (ECDC) and Global PPS study forms [13,27–29].

**Figure 1.**
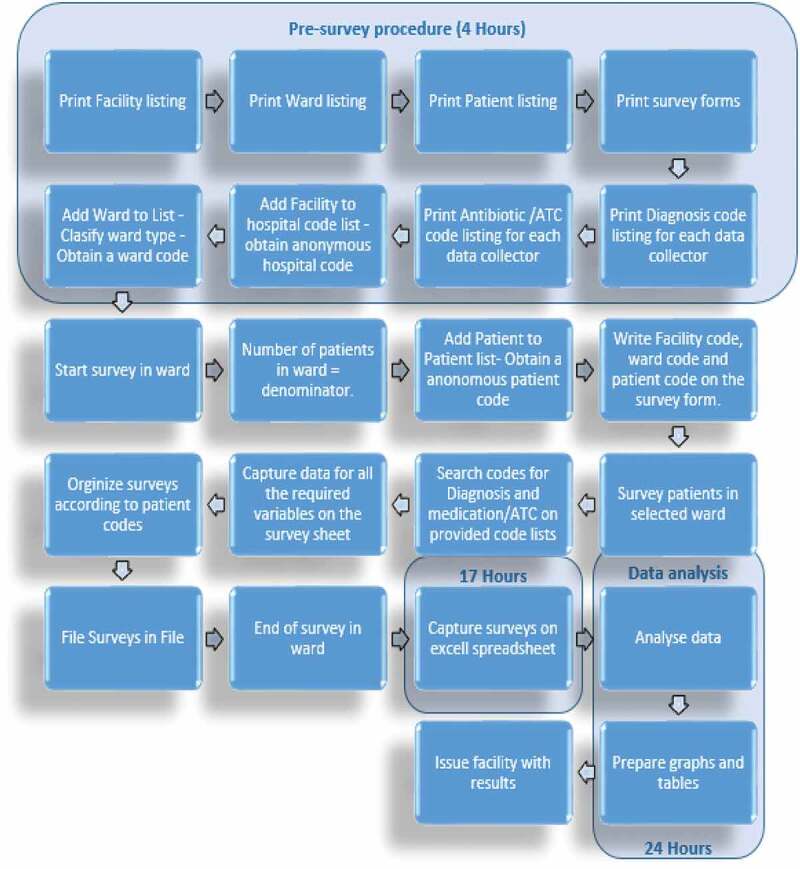
Workflow for paper-based data collection (based on 181 patient surveys)

Our initial study in South Africa demonstrated that it took considerable time for the data to be collected using the paper-based data collection instruments (DCIs) (Figure 1) [21]. The preparation for these DCIs involved prior printing of a facility listing to anonymously code each hospital, a ward listing to code, and classifying each ward according to a standard range of ward types, a patient listing to anonymously code each patient, and a survey form for each patient before starting the surveys. Overall, it took estimated 4 hours to perform the pre-survey procedure for a hospital with 34 wards. After the survey, it took an additional estimate of 48 hours to capture the data of 512 patients in Microsoft Excel^TM^ [21]. In view of this, an estimated time of 17 hours would be needed to capture 181 patient surveys irrespective of any data analysis time (see Section 2 – Methodology).

Consequently, the aim of this study was to develop and test a prototype app in a public hospital in South Africa to see if this would improve data collection in terms of the time associated with data collection and analysis versus the paper method and its usability.

## 2. Methods

The methodology involved a number of stages. These included (i) developing the app; (ii) testing and refining the app in a large public hospital in South Africa; (iii) analyzing the findings of the PPS; (iv) evaluating the time taken using the app and its ease of use

### 2.1 Developing the app

The variables included in the app were aligned principally to those included in the European Center for Disease Conrol (ECDC) and Global PPS studies with input from WHO [13,18,27,28]. However, as mentioned, adapted to include HIV, TB, malaria and malnutrition as confounders. This included assessing antimicrobial utilization by anatomical therapeutic chemical (ATC) classification (level 5) and their dose, frequency and route of administration [30]. Collected data on the treatment of the infection if an antimicrobial was prescribed included the antimicrobial prescribed, the rationale for its prescribing including whether this was recorded and whether for surgical or medical prophylaxis, whether the prescription was empirical or not, whether the antibiotic was administered or not and the route of administration, as well as other criteria to help assess the appropriateness of antimicrobial prescribing in this hospital. This was built on the findings of the previous paper-based study [21].

### 2.2 Testing and refining the app in a large public hospital in South Africa

This study involved PPS data being collected via mobile devices (e.g., iPhone and Samsung) over a two-week period by 15 trained data collectors using an early prototype of the app at the Dr George Mukhari Academic Hospital (DGMAH), which is one of the four academic hospitals in the Gauteng province. DGMAH is a 1,650-bed hospital with 28 clinical departments, 20 approved ICU beds, 60 high care beds, and 17 surgical theaters, providing services to an estimated 1.7 million people from the surrounding area. The app was subsequently tested across 13 wards at DGMAH. The wards were randomly selected and consisted of an adult ICU, three adult medical wards, three adult surgical wards, a hematology-oncology pediatric medical ward, two obstetrics and gynecology wards, a pediatric ICU, and two pediatric medical wards. Overall, 181 patients were surveyed.

Training on the use of the app was given over a two-day period via interactive PowerPoint presentations, and all users were instructed on the exact procedure for collecting the data. The first session over 2 hours on the first day primarily introduced the concept of using an app for collecting point prevelance data, with a brief overview of the data required for each field. The 2-hour session on the second day also included loading the app on each data collectors’ (personal) smartphone, and testing the technicalities. Some additional data were also provided, and the data collectors were asked to enter this onto the app to assess their understanding of both the app and the data to be collected. The training also included a pre-study session to determine their understanding of the app as a data collection instrument and given the opportunity to ask questions. In addition, multimedia demonstrations were also available as back-up after the training sessions (Appendix).

The app was designed to allow data collectors to collect data on a total of 35 variables (Table – Annexure A). The variables existed as multiple choice options as well as pre-programmed lists containing medication names, diagnosis and organism names, and some free text fields (Figure – Annexure B).

Data encryption was undertaken with both secure hash algorithm-256 (SHA-256) and Advance Encryption standard-265 (AES-256) [31], which are the strongest encryption technology currently available and the same level of encryption used by international banks [32]. The data backups consisted of both active and manual backups, and both the active backup and archives used the same encryption as the database. To minimize the risk of data mitigation failure, the data were stored in different geographic locations. The infrastructure is powered by Amazon Web Services (AWS), the industry leader in cloud services and is trusted by organizations such as DOW Jones, Pfizer and CDC in Atlanta. Only authenticated users have access to the database, various passwords protect the application, and passwords are protected by double encrypted password technology [31]. Furthermore, no patient sensitive data were stored directly within the app, and patient confidentiality was maintained throughout through the use of an anonymous coding system built directly into the application.

During the course of the PPS, the data collectors reviewed the patient medical records according to the PPS principles and collected the required data, with information subsequently entered directly into the app. There was no contact with patients or any healthcare professional interacting with the patient. Each data collector was added and linked to the facility and only had access to their own data. The survey would start by capturing the number of patients in the ward at the time of the survey as the denominator, typically 8 am, and end after all patients in the ward had been surveyed in line with the principle of PPSs [18,21]. Each patient in the ward was subsequently surveyed and the data captured directly into the app. The variables for diagnosis, organism and medication name/ATC code were entered via pre-programmed lists with search options to speed up data entry, with the search options building on the previous paper-based study as well as the PPS in Botswana [18,21]. The rest of the variables existed as multiple choice options with the data collector only selecting the correct option/s.

The app allowed users to view, edit, and delete the data they had entered, and was designed with the aim of making data entry less complicated and less time-consuming through an automated show/hide functionality to only display relevant fields based on the user’s previous inputs. Additional features that aimed to reduce the time taken to collect all pertinent data and the complexity of data entry included pre-programmed lists of medications, diagnoses and organisms to choose from.

Following data collection, the data were evaluated by the researchers for completeness and for obvious contradicting data entries such as an entry for ‘no consent’ when patients had not been asked for consent, patients on HAART being classified as not having HIV and where CST results were captured but data collectors indicated that blood cultures had not been taken. These contradicting entries were further assessed to determine whether the app could be further refined to eliminate these errors in the future. In additon, if further training was needed if it proved difficult to refine the app. Each data field was also subsequently checked for missing data and blank spaces alongside any contradictions in data entries.

### 2.3 Analyzing the findings of the PPS

The raw data was exported as a .csv-file into Microsoft Excel^TM^ spread sheets and evaluated by the researchers in Microsoft Excel^TM^ to ensure no data loss had occurred and that the file format would be suitable for statistical analysis. The findings were subsequently analyzed using SPSS Version 8.0 for Windows in consultation with a statistician. Descriptive statistics were performed on the retrieved data.

The findings from the PPS study were subsequently compared with the findings from the initial paper-based study in the same hospital [21] to help ensure the app collected all appropriate data.

### 2.4 Evaluating the time taken using the app and its ease of use

The workflow was evaluated afterward by comparing the processes between the paper-based DCI (Figure 1) and the app-based systems.

The usability of the app was also evaluated based on user feedback from a usability questionnaire. The questionnaire was developed using a set of questions based on the experience of the coauthors (ML and DK), which included questions on the perceived usefulness of the app and the time taken to complete each patient (Box 1). The questions were based on the Technology Accepted Model [33], with the data collectors responding to each statement based on a 4-point rating scale ranging from strongly agree to strongly disagree [25]. The same data collectors who had used the paper-based DCI for the initial PPS study were the ones undertaking the data collection using the app to enable meaningful comparisons.

**Box 1.** Content of the questionnaire to assess the usability of the developed app

The app was easy to use I think that I would need the support of a technical person to be able to use the app I found the app unnecessarily complex I found the various functions in this system were well integrated I thought there was too much inconsistency in this system I would imagine that most people would learn to use this app very quickly I found the app very difficult to use I felt very confident using the app I needed to learn a lot of things before I could get going with this app It enables me to accomplish task more quickly compared to the forms Overall, I find this product useful in PPS surveys How much time did you take to survey one patient file with the app How much time did you take to survey one patient file with the form

### 2.5 Ethical considerations

Ethical approval was received from the Sefako Makgatho University Research Ethics Committee (SMUREC/H/210/2016:PG). Additionally, permission was obtained from the Chief Executive Officers of DGMAH. Patient confidentiality was maintained at all times with a unique subject number allocated to each patient’s record to assure anonymity.

There was no contact with patients; consequently, no patient consent was needed to review the data, with all records anonymized.

## 3. Results

### 3.1 Antimicrobial utilization patterns

As mentioned, 181 patient files were reviewed from 13 wards of whom 151 (83%) were adults and 100 (55%) female. The median age (IQR) for males was 45.5 (25.5) years, and the median age for females was 42 (27) years. The overall prevalence of antimicrobial use was 44%. Whilst 38% (12 out of 31) patients in the adult surgical ward received antimicrobials, the prevalence was the higher (78%) in the pediatric medical wards. Less than half (42%) of the patients had not been hospitalized in the past 90 days and less than a third of those had used antimicrobials, with 30% of patient’s files having no history of prior hospitalization.

Amoxicillin and clavulanic acid were the most used antimicrobials followed by co-trimoxazole. A total of 54% of the antibiotics were initiated before taking a culture and 19% after taking a sample for a culture and sensitivity test (CST). The findings were similar to those from the paper-based study [21].

When the type of infection was not documented in the files, the data collectors typically used information from the physicians’ and nurses’ notes to check the onset of infection data and establish what type of infection this was. This type of practice will be the subject of future quality improvement programs in the hospital.

### 3.2 Refining the app

In total, 35 variables were included in the app, and no technical issues were reported on any of the variable fields during the data collection phase. However, an option to select ‘unknown’ for one of the variables was required during data collection.

During the evaluation, one medication name field was left empty and a total of four variables contained contradicting data. The incorrect data were either due to data collectors’ mistakes by choosing the wrong option provided or leaving a requested field empty. A number of these errors were eliminated by refining the app (Table 1).

**Table 1.** Variables for which incorrect data were observed

Variable	Number of incorrect entries	Refined in app
No consent – contradiction	6	No
HIV negative patients on HAART – contradiction	1	Yes
Antibiotic and route of administration – contradiction	2	No
Culture and sensitivity results ordered – unknown option	16	Yes
No culture taken, but culture results available in file – contradiction	2	Yes
Antimicrobial name for Surgical Prophylaxis – empty field	1	Yes

Although no consent was needed to survey the patient files at DGMAH, the data indicated that six patients had not given consent and no additional data were collected for these patients. This might either be as a result of data collectors not being familiar with the study protocol or data collectors not being attentive when entering the data. This was not refined on the app because the app is intended for use in other countries and hospitals where consent to survey patients’ files might also be required. However, a note was made to focus on this aspect more critically when other data collectors are being trained.

For the variable to determine if HIV patients were on HAART, a data collector indicated an HIV negative patient being on HAART. This error was eliminated by refining the app to only show the variable ‘on HAART’ if the patient is HIV positive.

For the variable to determine the route of administration of the antimicrobial, a data collector indicated in two patients that the route of administration was oral; however, the antimicrobial was ceftriaxone with currently no oral formulation for this medication. The app could not be refined to eliminate this error as some formulations might be available in different dosage forms in different counties.

However, the data collectors can avoid this error by making sure they enter the correct information, and this again will be emphasized during the training of new data collectors.

For the variable ‘culture sensitivity test (CST) ordered’, there was no option to select ‘unknown’ in the app. Consequently, 16 results were captured as ‘no culture taken’ before initiation of the antimicrobials. This is noted and the addition of an ‘unknown’ option for this variable will now be included in the updated app.

Two of the antimicrobials were captured as initiated without taking any cultures and that no culture was taken even after initiation of the antimicrobials. Despite this, the data collectors selected the option that culture sensitivity results were available in the patient’s file. This error was eliminated by subsequently refining the app to hide the variable of results available or not if the data collector indicated that the CST was not ordered.

On entering the names of the antimicrobials used for prophylaxis, one field containing the name of the antimicrobial was empty. The app was subsequently programmed to ensure this field is compulsory to reduce the risk of missing data in the future.

Five of the patients were also captured as having home-based care/facility acquired infection and this information was perceived as difficult to assess if this information was not documented in the file. Again, this concern will be brought up in future training programs.

### 3.3 Work flow for data collection

In comparison with the paper-based DCI (Figure 1), the data collection via the app required, as mentioned, the loading of the facility onto the app with a unique hospital code automatically assigned to each new facility. It is estimated that the pre-survey procedure took 1 hour. The wards in the hospital were subsequently loaded onto the app, and classified according to the standard ward types provided as a drop-down menu in the app.

**Figure 2.**
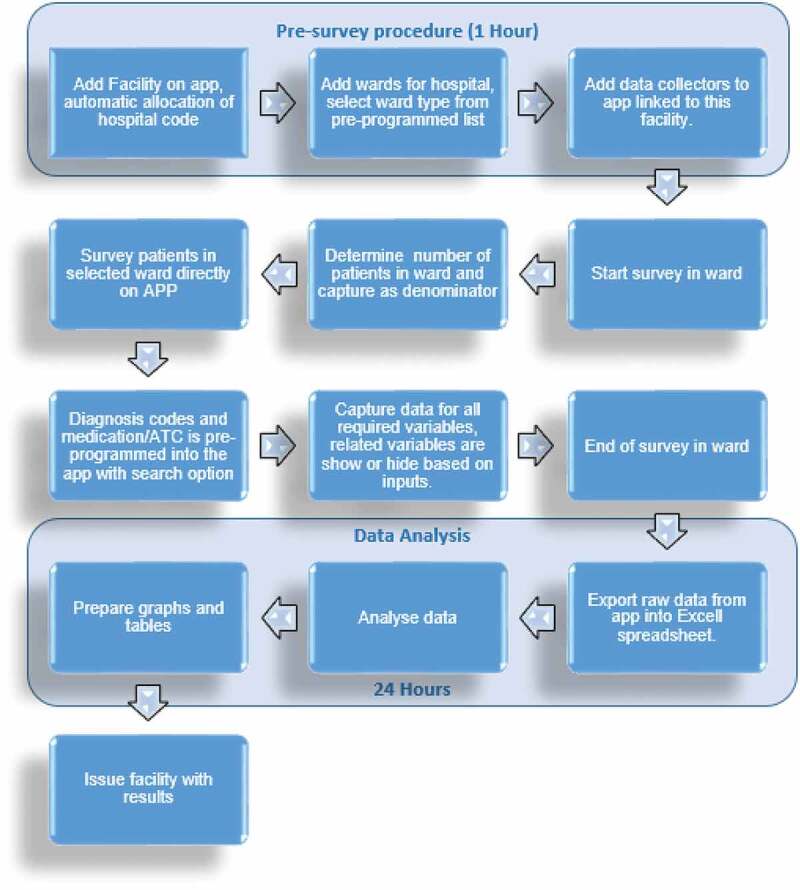
Process for app-based data collection tool

Figure 2 illustrates the workflow process for the app. The PPS data can subsequently be readily analyzed onto tables and graphs before issuing the facility with the findings for discussions regarding possible quality improvement programs. In comparison to the paper-based forms, any changes made to the app are immediately reflected in the app.

### 3.4 Data collection time and usability of the app

All 15 data collectors completed the questionnaire. The data collection time was measured through the questionnaire by requesting the data collectors indicate the time it took them to collect data from each patient file. Out of the 15 data collectors, 2 data collectors indicated that it took them 5 to 10 minutes to survey each patient file with the app, 10 indicated that it took 11 to 20 minutes, and three collectors indicated it took longer than 21 minutes (Table 2). The time taken for the paper-based tool was considerably longer (Table 2).

**Table 2.** Feedback on the usability of the APP (n = 15)

Questions	Strongly agree	Agree	Disagree	Strongly disagree
The app was easy to use	4	11	0	0
I think that I would need the support of a technical person to be able to use the app	0	0	15	0
I found the app unnecessarily complex	0	0	14	1
I found the various functions in this system were well integrated	2	12	1	0
I thought there was too much inconsistency in this system	0	1	9	5
I would imagine that most people would learn to use this app very quickly	0	15	0	0
I found the app very difficult to use	0	0	15	0
I felt very confident using the app	10	5	0	0
I needed to learn a lot of things before I could get going with this app	12	3	0	0
It enables me to accomplish task more quickly compared to the paper-based forms	12	2	0	1
Overall, I find this product useful in point prevalence surveys	11	4	0	0
How much time did you take to survey one patient file with the app	5 to 10 minutes	11 to 20 minutes	21 to 30 minutes	More than 30 minutes
Response from data collectors to this question	2	10	3	0
How much time did you take to survey one patient file with the paper based form	5 to 10 minutes	11 to 20 minutes	21 to 30 minutes	More than 30 minutes
Response from data collectors to this question	0	5	10	0

According to the data collector’s responses to the questionnaire, they were all confident in using the app and found the tool not at all complex to use. A majority of the data collectors (n = 14) found the functions of the app to be well integrated and consistent with only one disagreeing. A total of 11 strongly agreed and 4 agreed that the app could be useful in future PPS surveys. All the users agreed that they needed training with the app before they could use it (Table 2).

## 4. Discussion

The aim of developing an app was to provide an electronic data collection tool that was less time-consuming, and less costly than a paper-based DCI for PPS studies, to facilitate data entry, and to provide rapid feedback to all key stakeholders given current concerns with antimicrobial prescribing in hospitals in South Africa [20,21]. Several other studies that have compared electronic and paper-based data collection instruments have already shown that electronic data collection saves time and costs and is more reliable [25]. This study sought to build on these findings in a resource restricted African country as the need to collect and analyze antimicrobial usage data grows.

As seen, the findings regarding antimicrobial use in this study were similar to our first paper-based study in this leading hospital in South Africa [21]. Overall, the app allowed an appreciable amount of data to be collected within a short time period (Figure 2 versus Figure 1). A measure of data accuracy using errors showed that most errors can be avoided by having trained data collectors who are compliant to instructions and attentive to help reduce operator problems in capturing the data (Table 1) as well as subsequently refining the app to further reduce possible problems (Section 3.2). The pre-survey time was cut by 3 hours from 4 hours to 1 hour. In addition, the time taken to complete a patient was cut from typically 21 to 30 minutes down to typically 11 to 20 minutes (Table 2). This should be further improved with refinements to the app, as the data collectors become more familiar with it. Increased efficiency with data entering and validation was seen with the app as it automatically exported the data-file into Microsoft Excel^TM^ spreadsheets.

The operators indicated that the app was easy to use, well integrated, and they preferred it over the paper-based DCI methodology (Table 2). According to the comments, the majority of the users also found the use of electronic devices more convenient than numerous paper-based forms that needed to be printed and filed. The operators also felt that the forms were more labor intensive compared to the app. These findings were in line with other studies where participant’s acceptance of an electronic DCI was significantly more satisfactory compared to that of a paper based DCI [25].

As a result of this pilot study and its implications, further PPSs are underway among public hospitals in South Africa using an updated version of the app, and will be reported separately. During this research project, the app will be further refined if needed and additional reports given on its usability. The use of the app should also speed up the introduction and monitoring of pertinent quality improvement programs with real-time reports in hospitals. This will not only help to minimize the time until the facility can be issued with the ongoing results to plan future quality improvement programs if required, but also means that PPS studies can be undertaken more frequently in LMICs where there are serious manpower issues. This is important given the length of time generally between individual ECDC and Global PPS reports and the urgency surrounding AMR and inappropriate antibiotic use certainly among a number of African countries [11,13,17,34,35]. The data generated from this testing phase are already being used to inform an antimicrobial stewardship program at DGMAH, and we will be reporting on its outcome in future publications.

The addition of a timer into the app to accurately calculate the time between patient surveys is also highly recommended as this will ensure more reliable information on the time required to perform PPS surveys as well as be able to compare the various data collectors’ survey times to implement additional training as required. The data collectors could also potentially be given incentives to motivate them to collect data efficiently if this is a continuing concern. The costs for both DCI and the app can also be further evaluated to make accurate calculations on the total costs for each type of data collection tool to better plan for the future. We will now be working on this. Errors in the paper-based tools can also be further evaluated in order to make reliable comparisons of the data quality for both tools with the refined app if this is still needed to further demonstrate the overall efficiency with using the new app.

Finally, in considering the ability to export the app for use in other countries once further refined, the issue of the data repository for individual level data being hosted out of a country is an important consideration. This will require further information governance consideration if we wish to avoid duplication of app development across countries, and we will also be exploring this further.

We are aware that there are a number of limitations with this study. Firstly, the accuracy of the data captured is dependent on the accuracy of documentation in the files and the level of commitment of the data collectors. This is similar though to any PPS. Secondly, data were also only collected in a single facility, and we only used pharmacy students as data collectors. It may well be that data collection times could have been faster with trained pharmacists. Despite these concerns, we believe the app is the way forward for PPSs, and we are now taking this further in South Africa.

## 5. Conclusions

In conclusion, the app has sped up data collection in this PPS study as well as data analysis. However, it is recommended based on our findings that a real-time reporting section be developed in the app to speed up the introduction and monitoring of pertinent quality improvement programs with real-time reports, which is important given, as mentioned, the extent of inappropriate antibiotic use certainly among a number of African countries and the implications for rising AMR rate.

We believe based on our findings that the app is a potential tool to be used in future PPS studies as it has proven to be user-friendly and time saving. The app is currently being tested in a national PPS among public hospitals in South Africa and in all the provinces in South Africa, and we will be reporting the results in the future. The findings should provide additional guidance to further improve antimicrobial prescribing within hospitals in South Africa.

## Data Availability

The data that support the findings of this study are available from the corresponding author, BG, upon reasonable request.

## Declaration of financial/other relationships

Peer reviewers on this manuscript have no relevant financial or other relationships to disclose.

## Appendix

### Annexure A

**Table:**

	**VARIABLES:**
1	Patient consent
2	Patient age
3	Gender
4	Employment status
5	Transferred in from another facility or not
6	History of hospitalization in the past 90 days
7	History of antimicrobial use in the past 90 days
8	Was the patient on any catheter
9	Was the patient on any intubation
10	Does the patient have malaria
11	HIV status of the patient
12	Patient on HAART or not
13	Tb status of the patient
14	Was the patient malnourished or not
15	Diagnoses
16	Additional Surgery
17	Patient on any Antimicrobials
18	Antimicrobials used including dose
19	Hospital staff who prescribed the antimicrobial
20	Route of administration
21	Prescribed as Prophylaxis or Treatment
22	Was Prophylaxis Medical or Surgical
23	Duration of Antimicrobials for Surgical Prophylaxis
24	Type of Infection where Antimicrobials where prescribed for Treatment
25	If Culture and Sensitivity test (CST) was ordered before initiation of empiric antimicrobials
26	If Culture and Sensitivity (CST) result was available in the file
27	Name of organisms cultured
28	If treatment modified as per CST results
29	If IV antibiotics switched to Oral antibiotics if GIT was stable
30	Was this item prescribed from SA-EDL
31	If there were any missed doses
32	If missed doses were due to out of stock issues
33	If the item was written on the drug sheet
34	If item was prescribed from SA-EDL
35	If the item was prescribed in INN name
